# Rectal microbicides: clinically relevant approach to the design of rectal specific placebo formulations

**DOI:** 10.1186/1742-6405-8-12

**Published:** 2011-03-07

**Authors:** Lin Wang, Roger L Schnaare, Charlene Dezzutti, Peter A Anton, Lisa C Rohan

**Affiliations:** 1 Magee Womens Research Institute, Pittsburgh, PA, USA; 2 Department of Obstetrics, Gynecology & Reproductive Sciences, University of Pittsburgh, Pittsburgh, PA, USA; 3 Center for HIV Prevention Research, UCLA AIDS Institute, David Geffen School of Medicine at UCLA, Los Angeles, CA, USA; 4 Department of Pharmaceutical Sciences, School of Pharmacy, University of Pittsburgh, Pittsburgh, PA, USA

## Abstract

**Background:**

The objective of this study is to identify the critical formulation parameters controlling distribution and function for the rectal administration of microbicides in humans. Four placebo formulations were designed with a wide range of hydrophilic characteristics (aqueous to lipid) and rheological properties (Newtonian, shear thinning, thermal sensitive and thixotropic). Aqueous formulations using typical polymers to control viscosity were iso-osmotic and buffered to pH 7. Lipid formulations were developed from lipid solvent/lipid gelling agent binary mixtures. Testing included pharmaceutical function and stability as well as *in vitro *and *in vivo *toxicity.

**Results:**

The aqueous fluid placebo, based on poloxamer, was fluid at room temperature, thickened and became shear thinning at 37°C. The aqueous gel placebo used carbopol as the gelling agent, was shear thinning at room temperature and showed a typical decrease in viscosity with an increase in temperature. The lipid fluid placebo, myristyl myristate in isopropyl myristate, was relatively thin and temperature independent. The lipid gel placebo, glyceryl stearate and PEG-75 stearate in caprylic/capric triglycerides, was also shear thinning at both room temperature and 37°C but with significant time dependency or thixotropy. All formulations showed no rectal irritation in rabbits and were non-toxic using an *ex vivo *rectal explant model.

**Conclusions:**

Four placebo formulations ranging from fluid to gel in aqueous and lipid formats with a range of rheological properties were developed, tested, scaled-up, manufactured under cGMP conditions and enrolled in a formal stability program. Clinical testing of these formulations as placebos will serve as the basis for further microbicide formulation development with drug-containing products.

## Background

Over 33 million people were living with HIV worldwide in 2007, including 2.5 million people newly infected; 2 million people died of AIDS-related illnesses [1]. Receptive anal intercourse (RAI) is common in populations worldwide. In a multicenter AIDs cohort study, unprotected RAI, accounting for nearly all new HIV infections among the homosexual men enrolled in this study, is the riskiest sexual act that results in HIV infection [2]. According to Sigma Research in 2006, 89.3% of gay and bisexual men engaged in rectal intercourse at least once, 58.2% engaging in both insertive anal intercourse and RAI [3]. Despite knowing that condoms can prevent HIV transmission, large numbers of uninfected women and men practice sex without condoms [4] even with HIV-infected partners.

Rectal microbicides offer both primary protection against HIV in the absence of condoms and back-up protection when condoms fail [5]. Until now, microbicide research has been focused on vaginal use; the only Phase 1 rectal microbicide clinical trial uses a vaginal microbicide formulation [6,7]. However there are significant physiological differences between the rectal and vaginal compartments. Rectal epithelium is much thinner and more fragile, provides a greater surface area for infection, contains a greater number of CD4+ cells, and tissue adsorption is enhanced by lymphatic circulation. The relative enormity of the colon in comparison to the more limited size of the vaginal cavity is another problem. In addition, vaginal microflora is predominately Lactobacilli with acidic pH 4-5, while that of the rectum is predominantly anaerobic gram negative and gram-positive bacteria with minimal buffering capacity of neutral pH 7-8. Rectal toxicity associated with products containing nonoxynol-9 (N-9) [8-11] have been reported.

Personal lubricants are commonly used during anal intercourse [12,13]; some are not safe to use with fragile rectal tissue. Fuchs, et al. [14] evaluating the effects of marketed vaginal lubricants applied rectally found that hyper-osmolar gels caused rectal epithelial damage by inducing epithelia denudation and luminal secretion. Recent studies by Hendrix, et al. [15] have established the potential for semen and gel product distribution as high as the splenic flexure following simulated anal intercourse. These studies illustrate that the target surface for protection using a rectal microbicide may be quite large.

These considerations require site-specific formulation strategies for the development of safe and effective rectal microbicide products. Romano, et al. [16] have reviewed and outlined global strategies, Patton, et al. [17] has "outlined specific criteria" for preclinical rectal efficacy trials and Garg, et al. [18,19] have summarized preformulation and formulation from a pharmaceutics approach. However, to date, the ideal functional characteristics for a rectal microbicide have yet to be identified.

This study is part of a multi-center rectal microbicide development program designed in part to elucidate these functional characteristics [20]. The specific objective of this study is to develop a series of placebo formulations with a wide range of physical/chemical properties anticipated to be critical in rectal microbicide deployment and subsequently, in a separate human clinical study, evaluated for distribution and safety. The functional characteristics resulting from these two studies will form the basis for formulation development of a rectal microbicide for administration of water soluble or water insoluble active antiviral drugs, as single entities or, in combination.

## Results

### Baseline Data for Rectal Lubricants

Since personal lubricants are widely used during RAI, a series of marketed products were evaluated to identify a baseline of physical characteristics that might contribute to the placebo design. A sampling of the lubricant products was obtained on the open market covering the three basic types of lubricants, aqueous based, lipid based, and silicone based; these are listed in Table 1. Nine lubricants were evaluated for physical properties.

**Table 1:** Properties of selected commercial rectal lubricants

			Osmolality^a^	Viscosity^b^		Gel strength^a^	Adhesion^a^	Condom Compatibility^d^			
			
Product	Type	pH	mmol/kg	cps		mN	mN•sec	Lubricated		Non-lubricated	
							
				25°C	37°C			15 Min	30 Min	15 Min	30 Min
**Water based**											
Astroglide	Liquid	4	6110 ± 90	204.08	180.5	<4.9	NR^c^	39.9↑	42.5↑	49.9↑	11.0↑
Anal Lube	Gel	5.5	3530 ± 20	6700	6190	14.9 ± 0.5	196.0 ± 19.6	22.4↑	17.1↑	40.3↑	42.0↑
ID Glide	Gel	5.2	3150 ± 2	751	601	<4.9	NR	26.8↑	33.5↑	51.3↑	49.8↑
K-Y Jelly	Gel	4.5	2510 ± 26	7810	6560	14.9 ± 0.3	215.6 ± 19.6	52.7↑	46.6↑	49.3↑	34.2↑
**Lipid based**											
ID cream	Cream	NR	NR	1810	212	39.2 ± 2.0	42.1 ± 2.9	-45.0↓	-48.5↓	-25.9↓	-37.1↓
Boy Butter	Cream	NR	NR	4840	2600	34.3 ± 2.9	59.8 ± 2.9	20.4↑	25.2↑	40.2↑	42.7↑
Elbow Grease Original	Cream	NR	NR	5790	906	98.0 ± 9.8	58.8 ± 9.8	13.9↑	4.9↑	40.9↑	40.8↑
**Silicone based**											
Gun Oil	Liquid	NR	NR	175	138	NR	NR	10.5↑	15.8↑	20.9↑	22.5↑
Wet Platinum Premium	Liquid	NR	NR	145	113	NR	NR	13.4↑	-18.6↓	7.8↑	7.4↑

				Water (negative control)				22.3↑	20.2↑	14.8↑	15.3↑
				Mineral oil (positive control)				-46.6↓	-59.2↓	-51.1↓	-56.3↓

^a ^Reported as average (n = 3) of triplicate measurements

^b ^Reported as average of duplicate measurements.

^c ^NR, testing not required

^d ^Condom compatibility is expressed as percent change in puncture strength of treated vs untreated condoms

↔ No significant difference (p = 0.05).

↑ Treated sample significantly greater than untreated (p = 0.05).

↓ Treated sample significantly less than untreated (p = 0.05).

The rheological profiles of all the semisolid lubricants were non-Newtonian; the aqueous semisolids were pseudoplastic (shear thinning) while the lipid based semisolids were pseudoplastic with thixotropy. The liquid lubricants, both silicone and the aqueous based, all exhibited Newtonian behavior. On a comparative basis, viscosities of the lubricants (Table 1) ranged from 145 cps to 7810 cps at 25°C, 751 to 7810 cps for aqueous based lubricants, 1810 to 5790 cps for lipid based lubricants and less than 200 cps for the silicone based lubricants. The viscosity of the aqueous and the silicone lubricants were lower at 37°C than at 25°C but this was not significant. However, the lipid based semisolid lubricants, exhibited a highly significant temperature effect with up to a 9 fold decrease in viscosity between 25°C and 37°C.

The osmolalities of the aqueous lubricants varied from 2510 ± 26 to 6110 ± 90 mmol/kg, all of which were highly hyperosmolar. The pH values of the aqueous lubricants range from 4 to 5.5.

Condom/lubricant compatibility using the puncture strength test showed that in both groups of lubricated and non-lubricated condoms, water had a strengthening effect (p = 0.05) while mineral oil deteriorated condom integrity significantly (p = 0.05) overtime. The four aqueous gel lubricants tested, Astroglide (BioFilm, Inc., Vista, Ca), Anal Lube (California Exotic Novelties LLC., Chino, CA), ID Glide (ID Lubricants, Notts, NG UK), and K-Y Gelly (Personal Products Company, Skillman, NJ), had a strengthening effect on both lubricated and non-lubricated condoms when compared with the negative control; the effect was either equivalent to the control at 15 minutes or significantly increased puncture strength at 30 minutes. The lipid lubricants showed varied results. ID cream (ID Lubricants, Notts, NG, UK) showed the same degree of deterioration as mineral oil; however, the other two lipid based lubricants, Boy butter (Eyal Feldman, BBL LLC, CA) and Elbow grease (B. Cumming Company, Sun Valley, CA) were compatible with the condoms. For two silicone based lubricants, Gun oil (Empowered Products, Inc., Las Vegas, NV) and Wet Platinum (WET International, Valencia, CA), the effect on puncture strength was somewhat strengthening but less than water with the latter exhibiting a time dependent effect on lubricated condoms.

### Initial Placebo Design Parameters

Two fundamentally different approaches to establishing an effective rectally administered microbicide are possible; creating a formulation that spreads easily and coats all tissue surfaces of the rectum and distal colon rapidly prior to RAI or creating a deformable, erodible barrier that would remain relatively localized at the administration site until distributed by rectal intercourse. Accepting this premise and taking the results from the personal lubricants into account, we suggest that both aqueous and non-aqueous based formulations could be acceptable with target viscosities of < 200 cps and >5000 cps for the fluid and gel formulations, respectively. Aqueous formulations would be iso-osmotic with a pH of 7 and all formulations would be compatible with latex condoms. This suggests the following categories of formulations:

I. Aqueous Formulations:

a. Fluid - easily spreadable

b. Gel - erodible

II. Lipid Formulations:

a. Fluid - easily spreadable.

b. Gel - erodible

Fluid formulations would have a viscosity consistent with rapid rectal/colonic distribution while the distribution of gel formulations would be instigated by rectal intercourse.

### Aqueous Placebo Design

Prototype aqueous formulations were prepared with one of two polymers, which represent a range of chemical structures, as the viscosity or gelling agent, carbopol 974, poloxamer 407. Formulations ranging from fluids to gels were created by varying the concentration of polymer and were placed on stability at ambient temperature and 40°C. Viscosity, visual appearance and pH were followed over a period of 3 months.

### Lipid Placebo Design

The development of the lipid formulations presented a greater challenge considering the lack of lipid formulations in the vaginal or rectal market. Binary mixtures of lipid solvents and lipid gelling agents were screened for compatibility; the results of which are summarized in Table 2. Lipid solvents were selected to represent different chemical classes: triglycerides, fatty acid esters and POE fatty acid esters. Silicones, although not chemically lipids, were included as common non-aqueous lubricants. Lipid gelling agents were selected on a similar basis: fatty alcohols, fatty acid esters, glycerol fatty acid esters and POE fatty acids.

**Table 2:** Compatibility of binary lipid solvent/lipid gelling agent mixtures

	Gelling agent						
	
Solvent	Stearyl alcohol	Glyceryl tribemate	Myristyl myristate	Cetyl esters	Cetyl alcohol	Hydrogenated palm/palm kernel oil PEG-6 esters	Glyceryl stearate PEG-75 stearate
Olive oil	-	B1^a^	C1	-	-	-	-
Capric/Caprylic triglycerides	-	B2^a,b,d,e^	C2^a^	-	E2	F2^a^	G2^a,b,c,d,e^
Ethyl oleate	A3	-	C3^a^	D3	E3	F3^a^	G3^a^
Isopropyl myristate	A4	-	C4^a,b,c,d,e^	D4	E4	F4	G4^a^
Caprylocaproyl macrogol-8 glycerides	A5^a^	B5	C5	D5	E5^a^	F5^a,b,d,e^	G5
Silicone oil (350-500cs)	A6	B6	-	-	E6	F6	G6^a,b,d,e^
Cyclomethicone	A7	B7	-	-	E7	F7	G7

^a ^Potential miscible combinations used for further study. Miscible combinations can be obtained from stage of liquid to semisolid by varying the concentration level of gelling agent from 0-40%.

^b ^Stable combinations at least at one concentration level of gelling agent at room temperature

^c ^Stable combinations at least at one concentration level of gelling agent at both room temperature and 40°C

^d ^Rectal tissue explant toxicity were evaluated and showed nontoxic

^e ^Condom compatibility studies showed compatible with both non-lubricated and lubricated condoms

- Not tested

All others: incompatible combinations. Two phases upon made or short term storage at room temperature.

Binary mixtures were prepared by fusion at concentration levels of gelling agent (generally from 0.5% to 40%) to achieve a viscosity range from fluid to gel and placed on storage at 22°C and 40°C. An antioxidant was added to mixtures containing unsaturation, i.e. liquid fatty acids or triglycerides. Viscosity and visual appearance were followed over a period of 3 months. Compatibility was defined as complete miscibility during preparation and the absence of phase separation or precipitation during storage at either ambient or 40°C. As shown in Table 2, 14 out of a total of 36 mixtures tested were initially compatible; however, only two were stable enough to be considered further.

### Final Placebo Selection

Following the three month stability period formulations were evaluated for physical appearance and stability leading to the selection of the final four placebos listed in Table 3. The selection process also considered the ease of manufacture anticipating eventual scaling up to clinical size batches. An effort was also made to select formulations composed of a range of chemical structures, i.e., no duplication of solvents or gelling agents.

**Table 3:** Formulas for the four rectal microbicide placebos

	Fluid		Gel	
	
	Ingredient	Weight(%)	Ingredient	Weight(%)
	Poloxamer 407	15	Carbopol 974P	0.5
	Glycerin	2	Glycerin	2.5
**Aqueous**	Methylparaben	0.18	Methylparaben	0.18
	Propylparaben	0.02	Propylparaben	0.02
	Trisodium Citrate Dihydrate	0.3	Disodium EDTA	0.1
	Purified water	82.5	Sodium Hydroxide solution (18% w/v)	1.15
			Purified Water	95.55
				
		100		100

	Isopropyl Myristate	5	Glyceryl Stearate and PEG-75 Stearate	25
**Lipid**	Myristyl Myristate	95	Caprylic/Capric Triglyceride	74.9
			Vitamin E Acetate	0.1
				
		100		100

### Placebo Properties and Stability

The physical properties for the final four placebo formulations are summarized in Table 4; the stability data for 12 months at 25°C/60% RH and 6 months at 40°C/75% RH were all within testing specifications. Considering FDA guidelines regarding stability predictions, the 40°C data would support a shelf life in excess of two years [21].

**Table 4:** Physical properties of the four rectal microbicide placebos

		Viscosity^a^		Gel Strength ^b^	Adhesion^b^	Osmolarity		Condom Compability (puncture strength)				
								
Product	Appearance	cps					pH	Lubricated		Non-lubricated		Standard ASTM method
					
		25°C	37°C	mN	mN•sec	mmol/kg		15 Min	30 Min	15 Min	30 Min	
Lipid Gel*	White, creamy, rigid semi-solid	5634	2719	196.0 ± 2.0	127.4 ± 13.9	NR	NR	0.95 ↑	-6.1↔	32.8 ↑	25.8 ↑	Fail
Lipid Fluid*	Clear, colourless, easily spreadable liquid	5	4	NR^c^	NR	NR	NR	23.0 ↑	23.6 ↑	26.5 ↑	25.0 ↑	Fail
Aqueous Gel*	Transparent colourless gel	5613	5225	21.3 ± 0.2	31.9 ± 7.8	324 ± 15	7.2	33.8 ↑	30.1↑	18.8 ↑	27.5 ↑	Pass
Aqueous Fluid*	transparent colourless liquid	94	4136	NR	NR	402 ± 8	7.0	39.9 ↑	32.9 ↑	21.0 ↑	22.3 ↑	Pass

				Water (negative control)				22.3 ↑	20.2 ↑	14.8 ↑	15.3 ↑	
				Mineral Oil (positive control)				-46.6 ↓	-59.2 ↓	-51.0 ↓	-56.3 ↓	

^a ^Reported as average of duplicate measurements.

^b ^Reported as average ± SD(n = 3) of triplicate measurements

^c ^NR, testing not required

*passed microbial limit tests

↔ No significant difference (t-test, p = 0.05).

↑ Treated sample significantly greater than untreated (t-test, p = 0.05).

↓ Treated sample significantly less than untreated (t-test, p = 0.05).

#### Rheological Profiles

**Figure 1:**
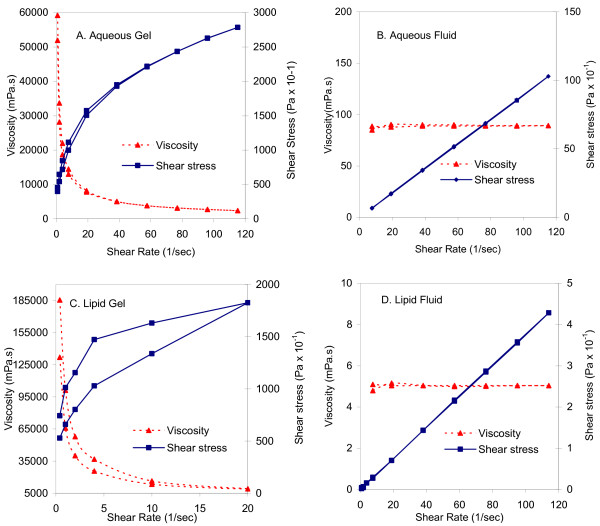
**Rheological profiles of the four rectal microbicide placebos**. The aqueous gel (1A) exhibits pseudoplastic behavior, the aqueous fluid (1B) exhibits Newtonian behavior, the lipid gel (1C) is thixotropic, and the lipid fluid (1D) is Newtonian.

The rheological profile of the aqueous gel placebo exhibits shear thinning and nonNewtonian pseudoplastic behavior (Figure 1A) with typical decrease in viscosity with an increase in temperature; similar to most of the commercial aqueous based semisolid personal lubricants evaluated. The lipid gel placebo (Figure 1C) exhibits similar shear thinning behavior with the addition of significant thixotropic or time-dependent character as evidenced by the hysteresis loop between the up curve and down curve.

The rheological profiles of the aqueous and lipid fluid placebos (Figure 1B and 1D) are Newtonian at 25°C, i.e. the viscosity is constant over the entire shear rate range. However, the aqueous fluid placebo exhibits a degree of nonNewtonian and shear thinning behavior at 37°C. The lipid fluid placebo, on the other hand, shows very little decrease in viscosity with an increase in temperature resulting in a formulation that maintains a relatively constant viscosity under anticipated use conditions.

#### Condom Compatibility

Condom compatibility was evaluated using a puncture strength test developed in house for rapid screening of a large number of formulations as well as standard ASTM method, which includes airburst volume, airburst pressure, tensile break force and elongation.

To develop the puncture strength method, a number of marketed product comprising water based, lipid based and silicone based products were evaluated (table 1). These studies confirmed that ID cream and Wet Platinum premium were incompatible with condoms as suggested on their marketed labels. The puncture strength test clearly differentiates between the aqueous and mineral oil controls (a range of 15% to 22% and -47% to -59% respectively, see Tables 1 and 4).

According to the puncture strength test, all aqueous placebo formulations show a degree of compatibility with lubricated and non-lubricated condoms. With respect to the lipid products, potential incompatibility with lubricated condoms was predicted. However, incompatibility with non lubricated condoms was not identified. With respect to the lipid gel studies with lubricated condoms (Table 4) the puncture strength test showed a 0.95% and -6.1% change compared to non treated lubricated condoms(for the 15 and 30 minute test conditions respectively), significantly different from the results of all the other cases.

Testing of the four placebo products using the ASTM method showed both of the lipid products failed to meet the acceptance criteria when tested with non-lubricated, silicone lubricated and aqueous lubricated condoms. The lipid formulations, however, did meet acceptance criteria with polyurethane condoms. The aqueous products were found to be condom compatible.

Although the puncture strength test did not completely agree with the standard ASTM condom test results for the two lipid formulations, it was able to identify condom incompatibilities in marketed products and suggested potential incompatibility with the lipid gel. This suggests that the puncture strength test can be a useful screening tool for rapid assessment of large numbers of formulations to eliminate those with highest potential for condom incompatibilities. However, full condom compatibility evaluation utilizing the ASTM method should be conducted on final formulations identified for advancement.

#### Rectal Toxicity Assessment

Using polarized colorectal explant cultures, N-9 was a positive control and showed a reduction of viability to 21.4% of the control, untreated explants. The lipid gel was 140% ± 64, the lipid fluid was 105% ± 26, the aqueous gel was 122% ± 66, and the aqueous gel was 122% ± 39 of the control, untreated explants. The non-toxic nature of the four formulations was confirmed by histology showing no alteration of the epithelium.

Using a rabbit rectal irritation model, the four placebo formulations had no significant damage at the rectal site of administration, no mortality occurred during the study and no clinical abnormalities or significant findings were observed. Gross pathology at necropsy provided no evidence for tissue damage or inflammation of the rectum or surrounding tissues. Collectively, these results support each other showing the safety of the rectal placebo formulations.

#### Microbiological Testing

All formulations passed the USP Microbial Limits test. In addition, the aqueous formulations passed the USP Antimicrobial Effectiveness Test.

#### Scale-up and GMP Manufacturing

The four placebo formulations were successfully scaled-up to a clinical batch size of 8 kg, were manufactured under cGMP conditions at DPT Laboratories, San Antonio, TX and placed on a formal stability program. The stability program will continue until the conclusion of planned clinical studies.

## Discussion

The initial placebo design attempted to consider two basic concepts, placebo performance as a drug delivery vehicle and the rectal delivery of a drug in such a formulation. The first concept was addressed by creating formulations that would include ready spreading and coating all tissue surfaces of the rectum and distal colon rapidly prior to RAI and alternately, formulations that would present a deformable, erodible barrier that would remain relatively localized at the administration site until distributed by rectal intercourse. With these concepts in mind and taking the results from the personal lubricants into account, representing a measure of practical acceptance, our work suggests that both aqueous and non-aqueous based formulations could be acceptable for the rectal administration of a microbicide.

The rheological properties designed in the placebos provide the clinician with a choice of physical performance. The aqueous gel placebo with shear thinning and nonNewtonian pseudoplastic behavior and typical decrease in viscosity with an increase in temperature compares with most of the commercial aqueous based semisolid personal lubricants evaluated. Viscosity recovery on cessation of shear induced by rectal intercourse would be instantaneous since no thixotropic behavior is observed. The lipid gel placebo, in contrast, exhibits similar shear thinning behavior with the addition of significant thixotropic or time-dependent character and as a consequence, viscosity recovery would be slow. Fluid properties produced by rectal intercourse would be maintained.

The Newtonian profiles of the aqueous and lipid fluid placebos with constant viscosity over the entire shear rate range would translate into efficient distribution on application. The aqueous fluid placebo would result in longer retention with the increase in viscosity as the formulation reaches body temperature. The lipid fluid placebo, on the other hand, shows very little decrease in viscosity with an increase in temperature resulting in a formulation that maintains a relatively constant viscosity under actual use conditions.

These rheological properties thus are anticipated to have the following behavior on rectal administration affected significantly whether rectal intercourse is present or not.

• The lipid fluid, being relatively thin with little temperature dependence, would distribute rapidly and efficiently after rectal administration.

• The aqueous fluid, with low viscosity initially for rapid distribution upon administration followed by increase in viscosity at body temperature would result in more localized retention.

• The lipid gel with high viscosity and being shear thinning permits distribution instigated by rectal intercourse and slow viscosity recovery permits continued distribution.

• The aqueous gel with relatively high viscosity and being shear thinning permits distribution instigated by rectal intercourse and rapid viscosity recovery permits localized retention following distribution.

In regards to the second concept, i.e., the rectal delivery of antivial drugs, accommodation of either water soluble or insoluble drug candidates was considered relative to bioavailability. Since current antiretroviral drugs being evaluated as microbicide candidates can be either water soluble as is the case for the reverse transcriptase inhibitor tenofovir or water insoluble as is the case for the nonnucleoside revese transcriptase inhibitors UC781 or dapivirine, both aqueous and non-aqueous or lipid formulations [22] need to be developed. This will provide for the opportunity to formulate either drug as a solution thereby providing a high concentration gradient for microbicide release [23]. Based on the four rectal placebo prototypes, for the aqueous formulations, hydrophobic or water insoluble drug candidates would be in suspension and hydrophilic or water soluble drug candidates would be in solution, while for the lipid formulations, hydrophobic drug candidates would be in solution and hydrophilic drug candidates would be in suspension.

The four placebo formulations are currently being evaluated in a human clinical study for mucosal toxicity, colo-rectal distribution, and participant acceptability. Clinical parameters evaluated will be related to product attributes such as viscosity, lipophilicity, spreadability, mucoadhesive characteristics. These correlations will ultimately lead to defining target specifications of rectal specific microbicide products.

## Conclusions

Four rectal microbicide placebo formulations were successfully designed, developed, tested and manufactured under cGMP conditions in anticipation of clinical evaluation in humans. The formulations have a wide range of hydrophilic characters and rheological properties and were shown to be non-toxic in both *ex vivo *and *in vivo *testing. It is anticipated that clinical testing of these formulations in humans will identify the critical formulation parameters controlling distribution and function in rectal administration. These results will serve as the basis for future rectal microbicide formulation design.

## Methods

### Materials

Carbopol 974P was obtained from Noveon (Lubrizol, Cleveland, Ohio), poloxamer 407 (Pluronic F127) from BASF (Florham Park, NJ), glycerylstearate and PEG-75 stearate (Gelot 64), caprylocaproyl macrogol-8 glycerides (Labrasol), and hydrogenated palm/palm kernel oil PEG-6 esters (Labrafil M 2130 CS) obtained from Gattefosse (Westwood, NJ), caprylic/capric triglyceride (Crodamol GTCC), isopropyl myristate (Crodamol IM), stearyl alcohol (Crodacol S-95 NF), cetyl esters wax (Crodamol SS NF), cetyl alcohol (Crodacol C-95 NF), glyceryl tribemate (Syncrowax HR-c), ethyl oleate (Crodamol EO), and myristyl myristate (Crodamol MM) obtained from Croda (Edison, NJ). All other chemicals were obtained from Spectrum Chemical Mfg. Corp. (New Brumswick, NJ) and were either USP or NF grade. Ingredients were GRAS [24,25] and/or commonly used in vaginal and rectal products [26].

### Preparation of lipid based placebo

Lipid gelling agents and lipid solvents (listed in table 2) were mixed and dissolved in a water bath held at 65°C -75°C depending on the melting point of the gelling agent. Additional excipient (Vitamin E, Vitamin E Acetate, Butylated hydroxyanisole or Butylated hydroxytoluene) was then added. The solution was cooled down to room temperature with stirring to achieve homogeneity.

### Preparation of aqueous based placebo

Different polymers (Carbopol or poloxamer) were evaluated as aqueous gelling agents.

For Carbopol based gels, 2.5% glycerin, 0.18% methylparaben and 0.02% propylparaben were dissolved in distilled water first by heating to 60°C. Carbopol was then added and dissolved in the solution by agitation at room temperature. Sodium hydroxide was added to induce Carbopol crosslinking. Finally pH was adjusted to 7 using sodium hydroxide or hydrochloric acid.

For poloxamer based gels, 0.18% methylparaben, 0.02% propylparaben, 2.5% glycerin and 0.1 M sodium citrate dehydrate buffer were dissolved in distilled water. The solution is then cooled to 4-8°C. After cooling the thermo reversible polymer, Poloxamer 407, was added. The mixture was then held overnight at 4-8°C for adequate polymer dissolution. Finally, pH was adjusted to 7 using sodium hydroxide or hydrochloric acid.

### Appearance

Samples were evaluated by visual inspection for phase separation, color, clarity, consistency and particulates in clear, glass scintillation vials.

### Viscosity

Rheological profiles were determined using a cone and plate viscometer (Brookfield HADV III+ and LVDV III ultra) recording shear stress over a range of shear rates at both increasing and decreasing shear rates, i.e. up curve and down curve, respectively. All viscosities reported are apparent viscosities calculated as the ratio of shear stress to shear rate. For comparison purposes, viscosities were calculated at a fixed shear rate of 10 rpm at 25 and 37°C.

### pH

The pH of the aqueous gel formulations was measured using a flat surface pH electrode (Beckman Coulter Futura Flat Bulk Combination pH electrode), that of aqueous fluid formulations using a glass pH electrode (Accumet pH electrode).

### Osmolality

The osmolality of aqueous formulations was measured using a vapor pressure osmometer (Model # 5520, Vescor).

### Gel Strength/Adhesion

For all semisolid formulations gel strength and adhesion were measured using the Texture Analyzer (TA.XT.Plus, Texture Technologies Corp.) [27,28] using a smooth surface and a ¼ inch ball probe. Gel strength is defined as the maximum force recorded during penetration (N) and adhesion is defined as the area under the force/distance curve on withdrawal (N•sec).

### Condom Compatibility Measurement

A method utilizing the Texture Analyzer was developed for the evaluation of condom/formulation compatibility. Briefly, 10 condoms were exposed to a formulation for a period of 15 and 30 minutes, washed using DI water, dried with a paper towel, and the puncture strength or breakpoint evaluated from the tensile/strain vs. force curve using a 1/8" ball probe.

In addition, a standard airburst test and tensile test for condom compatibility [29,30] (ISO-4074: 2002, ASTM D-3492) was contracted to a third party contractor who routinely conducts condom testing (Family Health International, FHI, Research Triangle Park, NC).

### Stability Studies

Formal stability assessments were performed according to ICH guidance [31]. Gel products were packaged in glass straight side jars, liquid products in glass Boston round bottles with poly-seal caps and stored at 25°C/60% RH or 40°C/75% RH. Testing intervals were 0, 1, 2, 3 and 6 months for the samples stored at 40°C/75% RH, and 0, 1, 2, 3, 6, 9 and 12 months for the samples stored at 25°C/60% RH. The testing included appearance, viscosity and microbial limit test [32] for all samples plus pH, osmolality and antimicrobial effectiveness test [33] for aqueous samples.

### Microbiological Testing

Microbiological testing was performed according to the USP30-NF25 General Chapters <61> Microbial Limit Tests (USP 30, page 83) for all formulations.

### Safety Testing Using *ex vivo *Polarized Colorectal Explant Cultures

#### Human Tissue

Normal human colorectal (IRB # 0602024) tissue was acquired from persons undergoing colorectal surgery for non-inflammatory conditions through IRB approved protocols. No patient identifiers were provided and all tissues collected were anonymized, de-identified, unlinking any patient ID to the investigators.

#### Explant Studies

Tissue toxicity of the test formulations was determined using a polarized explant human colorectal culture system [34,35]. Briefly, the explant was placed with the luminal side up in a transwell and the edges around the explant were sealed with Matrigel™ (BD Biosciences, San Jose, CA). The explants were maintained with the luminal surface at the air-liquid interface with the lamina propria resting on medium-soaked gelfoam. Cultures were maintained at 37°C in a 5% CO_2 _atmosphere.

Placebo formulations were placed undiluted on the apical side of the explants using untreated explants or a 1:5 dilution of 3% N9 gel as controls. The next day, explants were washed and viability was evaluated using the MTT [1-(4,5-dimethylthiazol-2-yl)-3,5-diphenylformazan] assay and histology.

### Rabbit Rectal Irritation Study

A ten day repeat dose toxicology study in New Zealand white rabbits following rectal administration of placebo formulations was performed by Pacific Biolabs (PBL, Hercules, CA). General morbidity and morbidity checks were performed twice daily for general health. Clinical observations were performed daily. Animals were observed for changes in their general appearance including, but no limited to, signs of dehydration, loss of weight, abnormal posture and hypothermia. Blood was collected from all animals just prior to necropsy on day 11 for hematology, coagulation, and clinical chemistry evaluations.

## Competing interests

The authors declare that they have no competing interests.

## Authors' contributions

LW carried out all the formulation compounding, testing and laboratory analysis, wrote reports and drafted the manuscript; RLS participated in project design, performed data and statistical analysis and helped draft the manuscript; CD conducted the colorectal explant experiments; PAA participated as MDP program principle investigator, oversaw project progress and reviewed and commented on the manuscript; LCR served as principle investigator for the MDP program formulation core, guided and participated in study conception, design, data analysis and drafting of the manuscript. All authors read and approved the final draft.
